# Neurological Manifestations of Severe SARS-CoV-2 Infection: Potential Mechanisms and Implications of Individualized Mechanical Ventilation Settings

**DOI:** 10.3389/fneur.2020.00845

**Published:** 2020-08-12

**Authors:** Denise Battaglini, Iole Brunetti, Pasquale Anania, Pietro Fiaschi, Gianluigi Zona, Lorenzo Ball, Daniele Roberto Giacobbe, Antonio Vena, Matteo Bassetti, Nicolò Patroniti, Angelo Schenone, Paolo Pelosi, Patricia R. M. Rocco, Chiara Robba

**Affiliations:** ^1^Department of Anesthesia and Intensive Care, San Martino Policlinico Hospital, IRCCS for Oncology and Neuroscience, Genoa, Italy; ^2^Department of Neurosurgery, San Martino Policlinico Hospital, IRCCS for Oncology and Neuroscience, Genoa, Italy; ^3^Department of Neurosciences, Rehabilitation, Ophthalmology, Genetics and Maternal and Child Health (DINOGMI), University of Genoa, Genoa, Italy; ^4^Department of Surgical Sciences and Integrated Diagnostics (DISC), University of Genoa, Genoa, Italy; ^5^Infectious Disease Unit, San Martino Policlinico Hospital, IRCCS for Oncology and Neuroscience, Genoa, Italy; ^6^Department of Neurology, San Martino Policlinico Hospital, IRCCS for Oncology and Neuroscience, Genoa, Italy; ^7^Laboratory of Pulmonary Investigation, Carlos Chagas Filho Institute of Biophysics, Federal University of Rio de Janeiro, Rio de Janeiro, Brazil; ^8^Ministry of Science, Technology, and Innovation, Brasília, Brazil; ^9^Rio de Janeiro Network on Neuroinflammation, Carlos Chagas Filho Foundation for Supporting Research in the State of Rio de Janeiro (FAPERJ), Rio de Janeiro, Brazil

**Keywords:** COVID-19, SARS-CoV-2, coronavirus, neurological manifestations, neurotropism

## Abstract

In December 2019, an outbreak of illness caused by a novel coronavirus (2019-nCoV, subsequently renamed SARS-CoV-2) was reported in Wuhan, China. Coronavirus disease 2019 (COVID-19) quickly spread worldwide to become a pandemic. Typical manifestations of COVID-19 include fever, dry cough, fatigue, and respiratory distress. In addition, both the central and peripheral nervous system can be affected by SARS-CoV-2 infection. These neurological changes may be caused by viral neurotropism, by a hyperinflammatory and hypercoagulative state, or even by mechanical ventilation-associated impairment. Hypoxia, endothelial cell damage, and the different impacts of different ventilatory strategies may all lead to increased stress and strain, potentially exacerbating the inflammatory response and leading to a complex interaction between the lungs and the brain. To date, no studies have taken into consideration the possible secondary effect of mechanical ventilation on brain recovery and outcomes. The aim of our review is to provide an updated overview of the potential pathogenic mechanisms of neurological manifestations in COVID-19, discuss the physiological issues related to brain-lung interactions, and propose strategies for optimization of respiratory support in critically ill patients with SARS-CoV-2 pneumonia.

## Introduction

**Figure 1:**
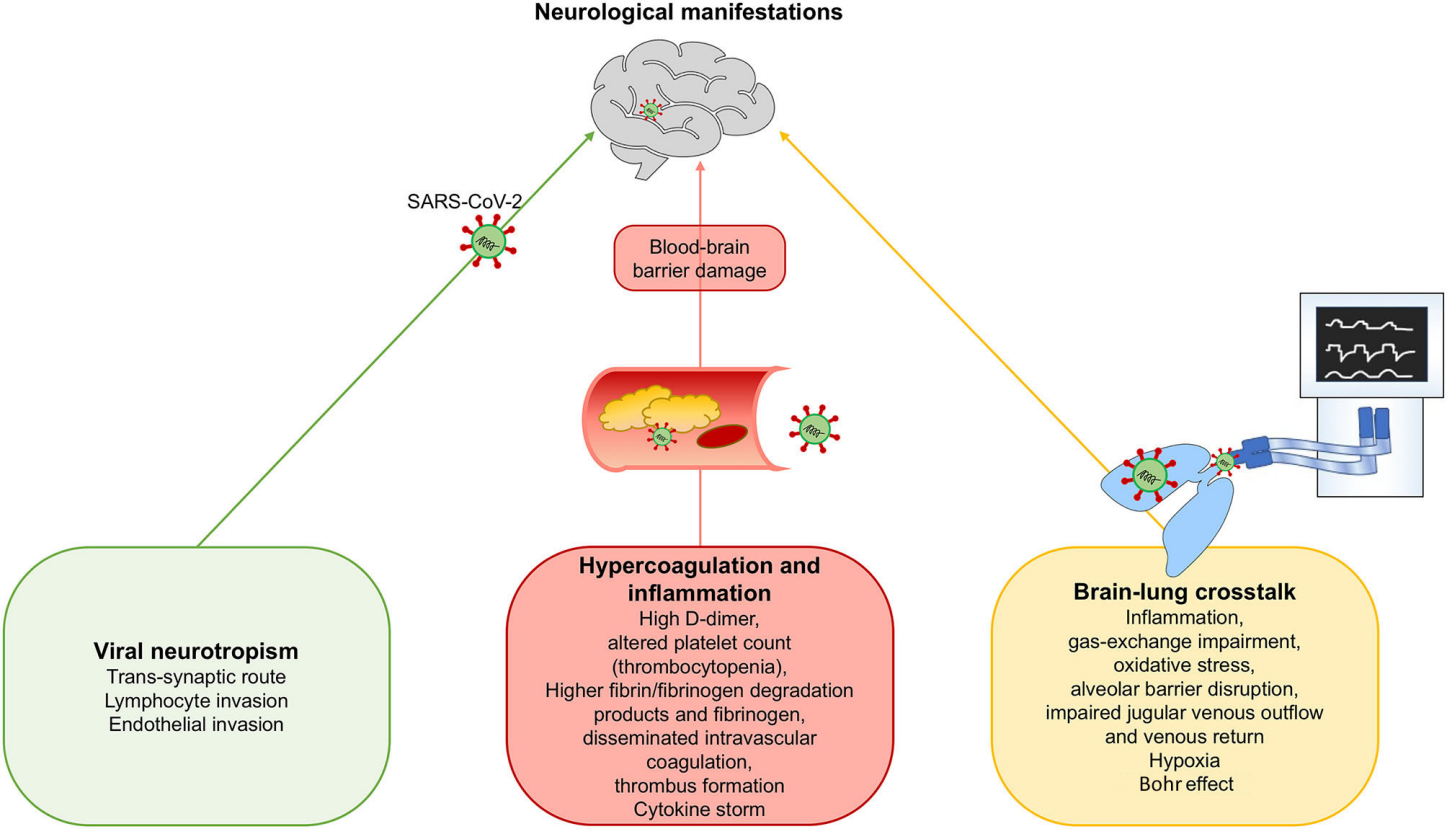
Proposed mechanisms for neurological manifestations in SARS-CoV-2 infection. We hypothesize three possible mechanisms for neurological manifestations in SARS-CoV-2 infection: (1) Viral neurotropism; (2) Hypercoagulation and inflammation, and (3) Brain-lung crosstalk.

In December 2019, an outbreak of disease caused by a novel coronavirus (2019 novel coronavirus, 2019-nCoV) was reported in Wuhan, China (1). On February 11, 2020, the novel virus was renamed the severe acute respiratory syndrome coronavirus-2 (SARS-CoV-2) by the International Committee on Taxonomy of Viruses, and on the same day, the disease it causes was named coronavirus disease 2019 (COVID-19) by the World Health Organization (WHO) (2). The rising number of daily confirmed cases globally led the WHO to characterize the outbreak as a pandemic on March 11, 2020 (3–8). The typical manifestations of COVID-19 include fever, dry cough, fatigue, and respiratory distress (9). Among patients with symptoms requiring hospitalization, 5–20% require invasive mechanical ventilation and admittance to an intensive care unit (10). COVID-19 is a complex, multisystem disease, perhaps best defined as a multiple organ dysfunction syndrome (MODS-CoV-2) (11) which includes neurologic manifestations (9). In a recent meta-analysis (12), headache was identified as one of the most common neurologic symptoms in the early stages of the disease (occurring in 3.5 to 34% of patients), followed by dizziness. More specific neurological manifestations were also observed, including impairment of smell, taste, or vision; limb weakness; acute cerebrovascular disease; and seizures. The causative mechanisms for neurological involvement in COVID-19 are still under-investigated because of a lack of prospective studies (12, 13). Furthermore, mechanical ventilation, commonly used in the management of COVID-19 patients, can itself induce an inflammatory response, causing distal organ failure. Thus, a complex cross-talk between the lungs and other organs, including the brain (14), may occur during severe COVID-19. Despite the paucity of evidence, there are three key hypotheses for the neurological manifestations of COVID-19 patients (Figure 1): (1) viral neurotropism; (2) a hyperinflammatory and hypercoagulable state; and (3) brain–lung crosstalk. While neuroinvasion may be restricted to most severe cases, other cases may be epiphenomena of systemic disease (11). The latter hypothesis is particularly interesting because it may be amenable to adjustment of ventilator settings to minimize lung and brain injury. Within this context, the aim of this manuscript is to provide an updated overview of the potential pathogenic mechanisms of neurological manifestations in COVID-19, discuss the physiological issues related to brain-lung interactions, and propose strategies for optimization of respiratory support in critically ill patients with SARS-CoV-2 pneumonia.

## Viral Neurotropism

### Pathogenesis

The coronaviruses are large, enveloped, non-segmented, single-stranded, positive-sense ribonucleic acid (RNA) viruses. Seven coronaviruses in two genera have been identified as possibly infectious in humans, of which SARS-CoV-1, Middle East respiratory syndrome (MERS-CoV), and SARS-CoV-2 can cause life-threatening respiratory failure (15, 16). Genomic and structural analyses have shown that SARS-CoV-1 binds to angiotensin-converting enzyme-2 (ACE2) receptors and transmembrane serine protease-2 (TMPRSS2) (17). MERS-CoV instead binds to dipeptidyl dipeptidase-4 (DPP4) receptors, which are mainly present on the epithelium of the lower respiratory tract, small intestine, liver, kidneys, and immune cells (18). ACE2 receptors are widely distributed in the lung alveolar epithelial cells, nasopharyngeal and oral mucosa, endothelium and vascular smooth muscle cells in the brain, vascular endothelium and smooth muscle cells of the liver, vascular and red pulp sinus endothelium of the spleen, and cytoplasm of distal tubules and collecting ducts in the kidney (17). However, binding to ACE2 and DPP4 receptors alone is not enough to make host cells susceptible to infection. Some human epithelial cells which overexpress these receptors are not infected, whereas other cells with lower expression of these receptors, such as central nervous system (CNS) cells, have shown SARS-CoV-1 and MERS-CoV infection (19). As with the other coronaviruses, the classical route of SARS-CoV-2 infection is the passage of infected droplets through the upper airway and binding to ACE2 receptors. Ocular transmission has also been proposed as a possible alternative route for SARS-CoV-2 infection, since the aqueous humor contains ACE2 receptors (20). SARS-CoV-2 enters the host cell by endocytosis. After viral uncoating, the virion is released, followed by translation, replication, virion assembly, and new virion coating, a process which induces programmed cell death (21). A cascade of cerebral involvement in SARS-CoV-2 infection has been proposed by many authors (22–24). Coronaviruses may pass from the systemic to the cerebral circulation by several routes. Trans-synaptic passage through infected neurons via the olfactory bulb has been demonstrated with other coronaviruses, which are able to invade peripheral nerve terminals and spread in a retrograde fashion through synapses into the CNS; neuroimaging evidence from COVID-19 patients suggests SARS-CoV-2 can do so as well. SARS-CoV-2 can also spread across the blood–brain barrier (BBB) by two distinct mechanisms: (a) leukocyte migration across the BBB (named the *Trojan horse mechanism*); and (b) sluggish movement of blood within the microcirculation, crossing the BBB by binding to endothelial cells (17). Infected leukocytes can bind to ACE2 receptors and cross the BBB, migrating into the CNS (22–26). Expression of ACE2 receptors has been demonstrated in neurons, astrocytes, oligodendrocytes, the motor cortex, the cytoplasm of neurons, and sympathetic pathways (22). Binding to ACE2 produces vasodilatation and counteracts inflammation, while binding to the Mas receptor exerts neuroprotective and cardioprotective effects (27).

### Experimental and Clinical Evidence

#### Trans-synaptic Spread

Literature from the previous SARS epidemic revealed that the virus primarily infects pneumocytes, but can also enter neuronal cells (28). Trans-synaptic spread has been demonstrated in experimental studies; in SARS-CoV-1 infected mice, extensive virus replication in brain cells was mediated by cerebral invasion through the olfactory epithelium (29). This has been also confirmed by another murine study with human coronavirus OC43 (30). In the clinical setting, SARS-CoV-1 genome sequences were detected in brain cells of infected patients by electron microscopy, real time-polymerase chain reaction (PCR), and light microscopy. Among brain areas, the thalami, cerebellum, white matter, and brainstem were primarily affected, with edema and scattered red degeneration of neurons (31). SARS-CoV-1 has been also detected in cerebrospinal fluid, probably reflecting spread through the BBB (29). Coronaviruses can also spread to the medullary cardiorespiratory center, which may at least partially account for the acute respiratory failure of SARS (32). Although previous literature on other coronaviruses clearly suggests neuronal involvement, data specific to SARS-CoV-2 are still limited; magnetic resonance imaging (MRI), autopsy findings, and brain biopsies should unravel the mystery. As with other coronaviruses, SARS-CoV-2 could potentially enter the nervous system through the olfactory bulb and spread to specific brain areas (33). This trans-synaptic spread theory is corroborated by multiple retrospective reports of anosmia and ageusia in COVID-19 patients (9, 29, 34). Most recently, anosmia and hyposmia were identified in 5.6% of 214 hospitalized patients (9), while 33.9% of 20 patients who completed a questionnaire experienced either olfactory or taste disorder and 18.6% experienced both (35). Smell and taste disorder were detected in 39.2% of 79 patients who were positive for COVID-19 PCR vs. 12.5% of 40 controls (adjusted odds ratio [OR] 21.4, confidence interval [CI] 95% 2.77–165.4, *p* = 0.003). Of these, 25 (80.6%) reported smell disorders and 28 (90.3%) reported taste disorders (34). A single center study on 1,480 patients with influenza-like symptoms revealed that smell and taste loss occurred in the majority of patients who tested positive for SARS-CoV-2, was significantly associated with COVID-19 (*p* < 0.001), and resolved after illness remission (36). A multicenter European study of 417 COVID-19 patients identified olfactory and gustatory dysfunctions as prevalent, early symptoms, which can indeed be used to identify SARS-CoV-2 infection (37). Finally, this hypothesis was confirmed *in vivo* by MRI evidence of cortical hyperintensity in the right gyrus rectus and olfactory bulb, suggesting viral invasion of the brain—although not all the patients who develop olfactory dysfunction present with abnormal brain imaging (38)—and in post-mortem brain MRI studies, which found olfactory bulb and tract impairment without brainstem involvement (39). This provides very compelling evidence of SARS-CoV-2 entry via the olfactory tract and subsequent spread to specific brain areas, although limited to isolated cases (2).

#### Endothelial and Lymphocyte Invasion

Electron microscopy studies have recently demonstrated that SARS-CoV-2 can cross the BBB by binding to endothelial cells (40). SARS-CoV-2 neurotropism was further confirmed in autopsies of infected patients who died of cardiorespiratory failure (>65 years old) and massive intracranial hemorrhage (younger). In both groups, all patients showed lymphocytic pan-encephalitis and meningitis (41), confirming the neurotropic hypothesis, perhaps guided by leukocyte invasion.

Irrespective of mechanism, neurotropism is thus clearly demonstrated. When brain involvement does occur, the presence and persistence of human coronaviruses in the CNS, as occurs in mice, can determine long-term neurological sequelae. Mice surviving acute coronaviral encephalitis exhibited long-term sequelae associated with decreased activity in an open field test and a reduced hippocampus, with neuronal loss in the Ammon's horn (CA)1 and CA3 areas (42). It has also been hypothesized that human coronaviruses may play a triggering role in long-term neurological conditions, such as multiple sclerosis. Although research has not led yet to a direct link to any specific virus, an association of coronaviruses with multiple sclerosis has been suggested (43, 44). A significantly higher prevalence of human CoV-OC43 was observed in the brains of multiple sclerosis patients than in controls (45). Moreover, during infection by human CoV-OC43 and CoV-229E, an autoreactive T-cell response directed to both viral and myelin antigens was discovered in multiple sclerosis patients, but not in controls (46, 47). This underlines the possibility that long-term infection of the CNS by human coronaviruses may play a role in the onset of multiple sclerosis-like demyelinating lesions, as reported during the COVID-19 pandemic (48). Evidence of CNS infection by SARS-CoV-2 has been associated with poor prognosis, worse clinical condition, and sudden death in COVID-19 patients (9). However, there is limited evidence to confirm this hypothesis, since the majority of observed cerebrospinal fluid (CSF) samples have been negative for SARS-CoV-2 infection (49, 50). This makes it difficult to confirm that neurotropism could be the main mechanism of neurological complications in COVID-19.

## Hyper-Inflammation and Hypercoagulability

### Pathogenesis

SARS-CoV-2 may pass across the respiratory epithelium and spread from the alveolar-epithelial barrier to the systemic circulation, enhancing the local inflammatory response (51) and producing a systemic “cytokine storm,” affecting other organs such as the brain (52). Furthermore, inflammation is one of the main mechanisms that trigger the coagulation cascade and promote hypercoagulability. In severe SARS-CoV-2 infection, recent findings suggest a key role of endothelial cells (ECs) in vascular dysfunction, immunothrombosis, and inflammation (53). Histopathological studies have provided evidence of direct viral infection of ECs, diffuse endotheliitis, and micro- and macrovascular thrombosis, both in the venous and arterial circulations. The pro-inflammatory cytokine storm, with elevated levels of interleukin-6 (IL-6), IL-2 receptor, and tumor necrosis factor (TNF)-α, could also participate in endothelial dysfunction and leukocyte recruitment in the microvasculature. COVID-19-induced endotheliitis may explain the systemic impaired microcirculatory function in different organs observed in COVID-19 patients. Next, we will discuss the role of hyperinflammation and hypercoagulability as potential mechanisms for secondary brain involvement in COVID-19.

On the immune side, after antigen binding to the host receptor, monocytes are activated, with the release of pro-inflammatory cytokines (such as MMP9, which increases BBB permeability, and TNF-α, which that increases expression of intracellular adhesion molecule [ICAM]-1 on endothelial cells). Infected and activated monocytes cross the damaged BBB, inducing the local release of pro-inflammatory cytokines and resulting in oligodendrocyte and neuronal damage. Coronavirus primarily infects monocyte-derived macrophages, which produce chemokines and then present CoV antigens to T-cells and other pro-inflammatory cells (51). Astrocytes may also release other chemokines that will recruit other leukocytes. This hyperactive neuroinflammatory response could induce immune-mediated neuropathology (2, 51). On the coagulation side, increased consumption and decreased production of platelets in the damaged lungs are all factors that can contribute to thrombocytopenia (54). As a consequence, it seems reasonable that infected patients are more prone to developing posttraumatic or spontaneous intracranial hemorrhage (55), as well as these alterations suggest a trend of SARS-CoV-2 infection to induce consumption coagulopathy, which, if unchecked, could lead to disseminated intravascular coagulation (DIC) and an unfavorable clinical course (56). In fact, viral infections may lead to sepsis, which represents the most common cause of DIC. DIC is determined by the release of injury-related cytokines, which activate monocytes and endothelial cells, leading to overexpression of tissue factors and secretion of von Willebrand factor. The presence of free thrombin in the circulation can activate platelets, stimulating fibrinolysis (57).

### Experimental and Clinical Evidence

#### Inflammation

Inflammatory involvement was recently confirmed by an experimental murine model of murine coronavirus (MHV-A59), which can enter the brain via intranasal or intracerebral exposure and whose virulence is mediated by cytokine secretion. In one experimental study, injection of mouse hepatitis virus (MHV), a member of the *Coronaviridae* family, into the murine CNS demonstrated that coronavirus infection elicits both innate and adaptive immune responses (58). The genomic RNA is then translated, replicated, assembled, and coated for future release and infection of other cells. As replication increases with the aid of macrophages, microglia, astrocytes, and oligodendrocytes, the virus can spread from the ependyma to the brain parenchyma. By this point, inflammation is established, and is followed by BBB damage and enhanced innate and adaptive immune responses (58). Immunofluorescence and immunohistochemistry revealed that microglia and astrocytes are involved in activation of the innate immune system of the brain, releasing cytokines that are involved in the pathogenesis of encephalitis (59). A study on human autopsy specimens showed that SARS-CoV-1 was able to infect brain tissue, with necrosis of neuronal cells and gliocyte hyperplasia. These studies suggested that neuronal involvement in SARS was characterized by a massive inflammatory process, especially with enhancement of monokine expression in gliocytes induced by interferon (IFN)-γ (60). An experimental study on bronchoalveolar lavage fluid (BALF) of COVID-19 patients identified that SARS-CoV-2 infection of the airway leads to pro-inflammatory cytokine and chemokine release. This enhances the interaction with receptors expressed on thoracic sensory neurons of the lung, thus causing the release of neuropeptides, followed by vasodilation, immune-cell recruitment, neurogenic inflammation, and potential pain. This mechanism could be theoretically involved in the hyperinflammatory state, which first involves the lung and then extends to the nervous system, with sensory neurons thus potentially acting as drivers of neurogenic pulmonary dysfunction (61). In a retrospective cohort cited above, severe patients were more likely to exhibit impaired consciousness and acute cerebrovascular disease than non-severe patients (*p* < 0.001 and *p* < 0.05, respectively). Severe patients also showed a more florid inflammatory response (higher white blood cell and neutrophil counts, lower lymphocyte counts, higher C-reactive protein levels) and higher D-dimer levels than non-severe patients, and developed more extensive multiple organ involvement (9). Acute necrotizing encephalopathy (ANE) has been related to a brain cytokine storm, which results in BBB disruption (62). ANE has been previously reported as a rare complication of viral infections such as influenza (62). Radiological findings from computed tomography (CT) scans and MRI in COVID-19 have been recently published (62). ANE was also identified in a patient with aplastic anemia (63). Non-contrast CT scan demonstrated bilateral symmetric hypoattenuation in the medial thalami with negative CT angiogram and venogram findings, while MRI showed bilateral hemorrhagic rims in the thalami, sub-insular regions, and medial temporal lobes. ANE usually presents a bilateral distribution, with predominance of lesions in the thalami, brainstem, cerebral white matter and cerebellum, which is consistent with the cerebral insults observed in COVID-19 (62). Studies have concluded that men and women might show different responses to COVID-19. Women seem to be less susceptible to viral infections than men overall. The presence of two X chromosomes influences immune regulatory genes to blunt the inflammatory response and increase levels of antibodies and cluster of differentiation (CD)4^+^T-cells, and consequently, promoting the expression of cytokines. Moreover, the X chromosome acts on other proteins and genes, including forkhead box (FOX)P-3, toll like receptor (TLR)-8, CD40L, and chemokine receptor (CXCR)3. Nevertheless, the increased susceptibility of women to autoimmune and auto-inflammatory disorders has to be taken into account (64). Coronavirus infection of the CNS has long provided a model for studying demyelinating diseases such as multiple sclerosis, vaccine design, and novel immunotherapeutic to limit virus spread (58). Hemophagocytic lymphohistiocytosis (HLH) is characterized by a severe dysregulation of T-lymphocytes, natural killer cells, and macrophages within the contest of cytokine storm and multiorgan failure, and represents a clear link between hyperinflammation and hypercoagulability (65). This condition has been described in patients with SARS-CoV-2 (1). HLH patients present with pancytopenia, coagulopathy, hepatic dysfunction, hypertriglyceridemia, and high ferritin levels (66).

#### Coagulopathy

**Figure 2:**
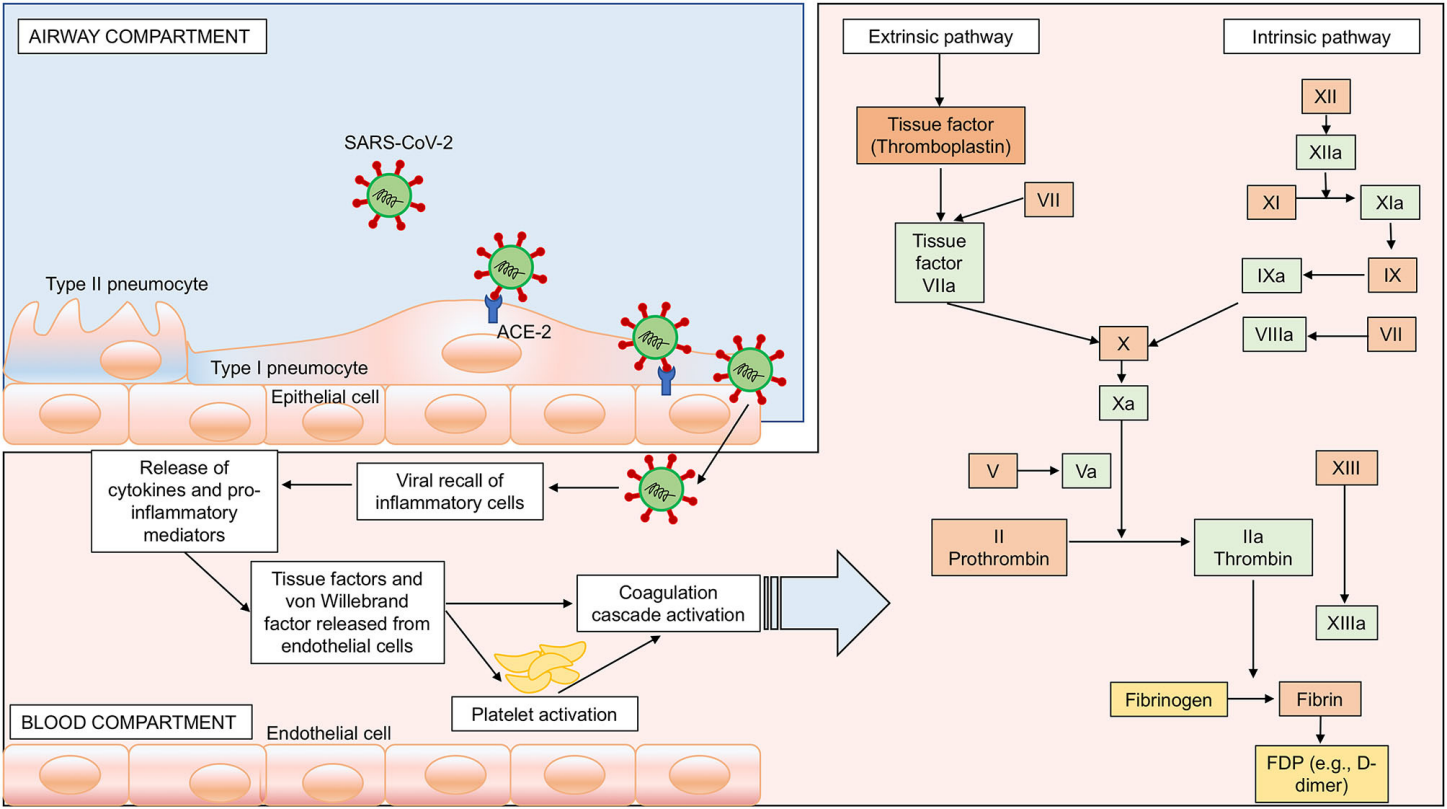
SARS-CoV-2-induced hypercoagulability. Passage of the virus from the airway to the systemic circulation is facilitated by the sluggish movement of blood within the microcirculation and subsequent binding of ACE-2 receptors, expressed on the capillary endothelium, followed by endothelial damage, enhanced inflammation, and hypercoagulability. In this figure, we represent the activation of both intrinsic and extrinsic coagulation pathways as a possible mechanism for hypercoagulability and potential brain damage. Intrinsic pathway: activation of factor (F) XIIa, followed by activation of FXIa and VIII. Extrinsic pathway: activation of FVIIa and tissue factor. Both pathways converge in the common pathway with activation of FXa, FVa, prothrombin into thrombin, fibrinogen into fibrin, and fibrin degradation products (FDP) such as D-dimer.

Neurological damage in COVID-19 patients may also be associated with coagulopathy. In a recent meta-analysis, Lippi et al. showed that low platelet counts are associated with poor prognosis in COVID-19 (67). As reported by Yang et al. (54), hematological changes were common in patients with SARS, most notably including lymphopenia and thrombocytopenia, through different potential mechanisms. Preliminary data from COVID-19 cohorts described a major impairment of blood coagulation and derangement of hemostasis in a large number of patients. Han et al. (68) studied alterations in blood coagulation parameters of patients with SARS-CoV-2 infection, observing lower antithrombin values and higher D-dimer, fibrin/fibrinogen degradation products, and fibrinogen levels. Tang et al. (56) observed high levels of D-dimer and fibrin/fibrinogen degradation products in all non-survivors, confirming activation of coagulation cascade and secondary hyperfibrinolysis. Within this context, the neurological manifestations associated with SARS-CoV-2 may be determined by a hypercoagulable state with high D-dimer levels. The association between ischemic stroke and high D-dimer levels has been previously described in the literature (69, 70). D-dimer elevation reflects ongoing thrombus formation, although it is also an acute-phase reactant that enhances the inflammatory process itself by stimulating monocyte synthesis and release of proinflammatory cytokines (e.g., IL-6), thus contributing to stroke occurrence and progression (71). Coagulopathy and antiphospholipid antibodies were found in patients affected by COVID-19. These findings were associated with both arterial and venous thrombotic events, including cerebral infarcts and limb ischemia. Patients presented with prolonged activated partial thromboplastin and prothrombin times, while two of three patients showed thrombocytopenia (72). Fourteen cases of stroke have been reported out of 214 patients in China (9). Likewise, MRI and CT scans revealed a high prevalence of stroke in COVID-19 patients (49, 73–75), including in patients younger than 50 years (76). The association between stroke and COVID-19 could be explained also by the fact that both diseases share the same risk factors such as hypertension and diabetes (77, 78), and by the pathological hypercoagulability state that characterize COVID-19. An association between high levels of D-dimer and intracerebral hemorrhage (ICH) was described in a prospective study carried out by Di Castelnuovo et al. (79), although a previous meta-analysis did not show a causal relationship (80). A recent meta-analysis by Zhou et al. (81), which included 13 studies on 891 patients with ICH, concluded that high levels of D-dimer were associated with an elevated risk of ICH. In fact, high D-dimer levels stimulate fibrinolysis with subsequent plasmin generation and microvascular lesions, which might cause the inhibition of hemostasis and a hypo-coagulable state, thus triggering cerebral hemorrhage (79). Moreover, an association between elevated D-dimer levels and large hematoma volume, intraventricular and subarachnoid blood extension, and early mortality has been reported in ICH (82). In summary, although literature is inconclusive concerning the relationship between COVID-19-related hypercoagulability and neurological complications, a possible correlation should be taken into account. Possible mechanisms for activation of intrinsic and extrinsic coagulation pathways, followed by inflammation by SARS-CoV-2 infection are proposed in Figure 2.

## Brain–Lung Crosstalk in COVID-19: an Underestimated Mechanism

### Pathogenesis

**Figure 3:**
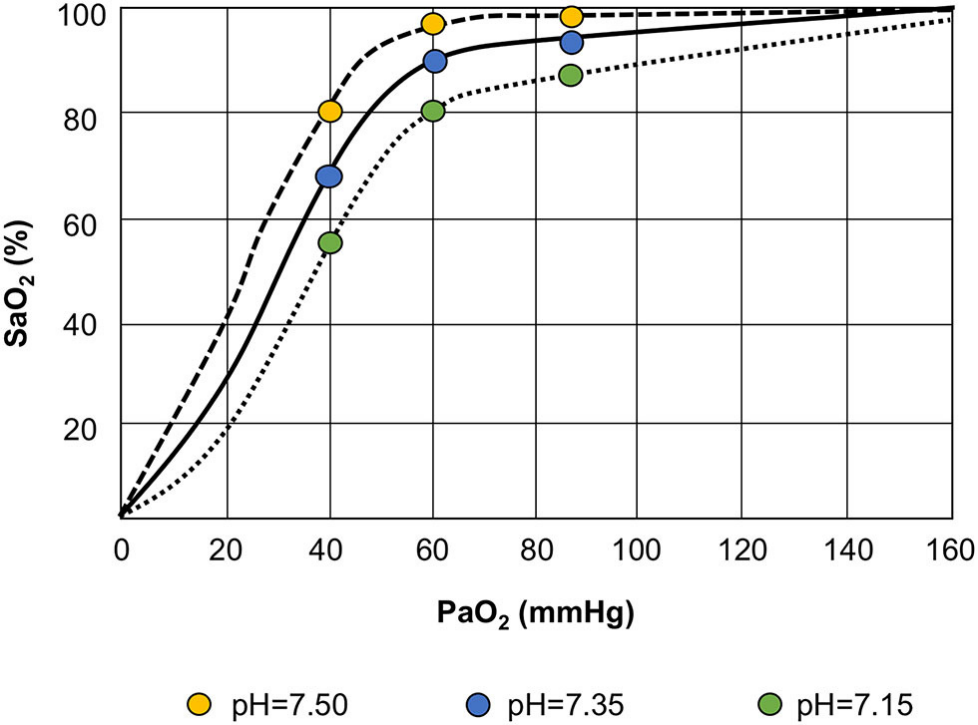
Bohr effect. The oxyhemoglobin dissociation curve is shifted to the left in response to respiratory alkalosis (lower PaCO_2_ and higher pH), with increased affinity of oxygen for the hemoglobin. Conversely, during respiratory acidosis (higher PaCO_2_ and lower pH), the alveolar oxygen tension and systemic saturation improve, thus reducing alveolar carbon dioxide tension, as explained by the Bohr effect: the higher the acidity, the more carbon dioxide is eliminated.

Brain–lung crosstalk and its implications for ventilator management are illustrated in Figure 3. The respiratory management of COVID-19 shares some characteristics with that of the acute respiratory distress syndrome (ARDS) (83), but different hallmarks must be considered and discussed. COVID-19 pneumonia is as a typical “pulmonary” ARDS (84). In experimental settings (85), “pulmonary” as compared to “extrapulmonary” ARDS is distinguished by increased alveolar–epithelial damage, more neutrophil cell infiltration and fibrinous exudate, increased collagen fibers in the alveoli and interstitium. In clinical studies, different radiological patterns have been identified, with different characteristics and responses to alveolar recruitment. In non-COVID patients, ARDS is characterized by interstitial and alveolar edema homogeneously distributed along the vertical gradient (86, 87), leading to collapse of the most dependent alveoli in the supine position. Regional perfusion follows a gravitational gradient (more perfusion in dependent lung regions), and severe hypoxemia is explained mainly by increased “true shunt” in atelectatic, dependent lung regions. Application of higher levels of positive end-expiratory pressure (PEEP) is associated with alveolar recruitment, improving respiratory mechanics and gas exchange. Thus, in classical ARDS patients, therapeutic maneuvers leading to improvement in gas exchange are associated with better lung aeration. Conversely, COVID-19 pneumonia is characterized by minimal interstitial and alveolar edema, alveolar cellular infiltration and necrosis, with alveolar consolidation and pneumolysis. Regional perfusion follows a non-gravitational gradient (more perfusion in non-dependent lung regions), with hyperperfusion of normally aerated and poorly aerated (“ground glass”) tissue, leading to major changes in ventilation-perfusion ratio. Additionally, perfusion in consolidated, dependent lung regions contributes to “true” shunt. Application of higher levels of PEEP does not recruit alveoli; instead, it leads to deterioration of respiratory mechanics, gas exchange, and hemodynamics. Thus, in COVID-19 patients, therapeutic maneuvers leading to improvement in gas-exchange are not associated with improved lung aeration, but rather with redistribution of regional perfusion (88). Interestingly, areas of hypoperfusion may occur in poorly aerated ground-glass areas as well as in non-aerated lung regions. This suggests that some hypoperfusion might be protective against further deterioration of ventilation-perfusion ratio as well as “true” shunt.

Three distinct radiological phenotypes of COVID-19 pneumonia have been described (79). Phenotype 1 is characterized by multiple, focal, overperfused ground-glass opacities, normal or high lung compliance, and severe hypoxemia, probably caused by low ventilation/perfusion and regional shunting. In this case, PEEP should be set according to the lowest driving pressure and/or minimal oxygenation, and inhaled nitric oxide might be useful. Phenotype 2 is characterized by an inhomogeneous and/or asymmetrical distribution of atelectasis, partial alveolar derecruitment, and/or consolidation with peribronchial opacities. In these cases, lateral or prone positioning might be helpful. Finally, phenotype 3 is characterized by patchy, ARDS-like diffuse lung infiltration, with a mixed pattern of overperfused, normally aerated and ground-glass areas as well as hypoperfused, non-aerated lung regions with low compliance. In this setting, mechanical ventilation should follow standard protective ventilatory strategies used for ARDS, with minimal PEEP, prone positioning, and escalation to extracorporeal membrane oxygenation (ECMO) as needed. In all cases, possible microthrombosis and multiorgan failure must be considered.

**Figure 4:**
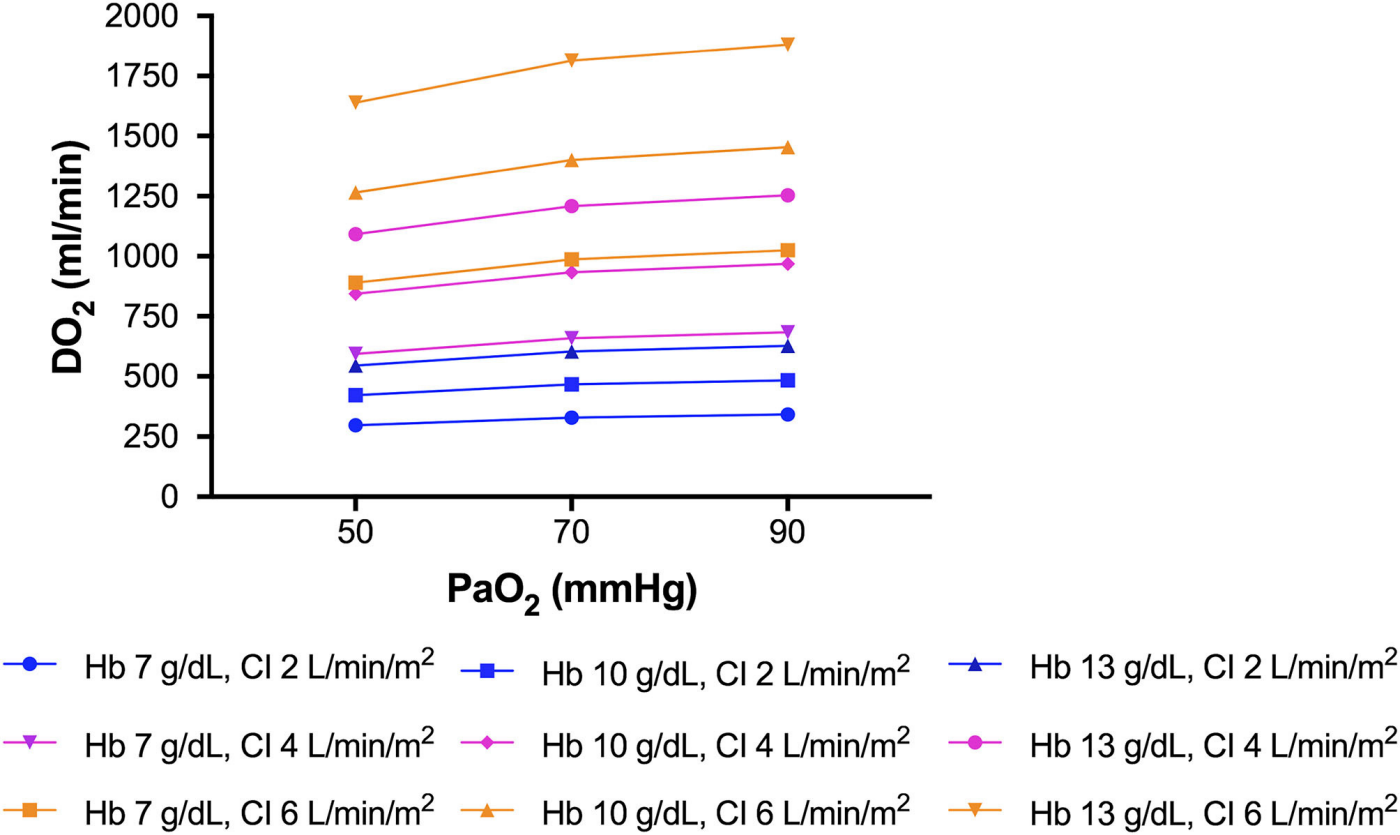
Improving oxygen delivery to the brain. Raising hemoglobin and cardiac output should be considered for improving oxygen delivery, especially in COVID-19 phenotype 1. This figure represents different delivery of oxygen (DO_2_) at a fixed cardiac output, by changing hemoglobin, or at fixed hemoglobin, by changing cardiac output.

**Figure 5:**
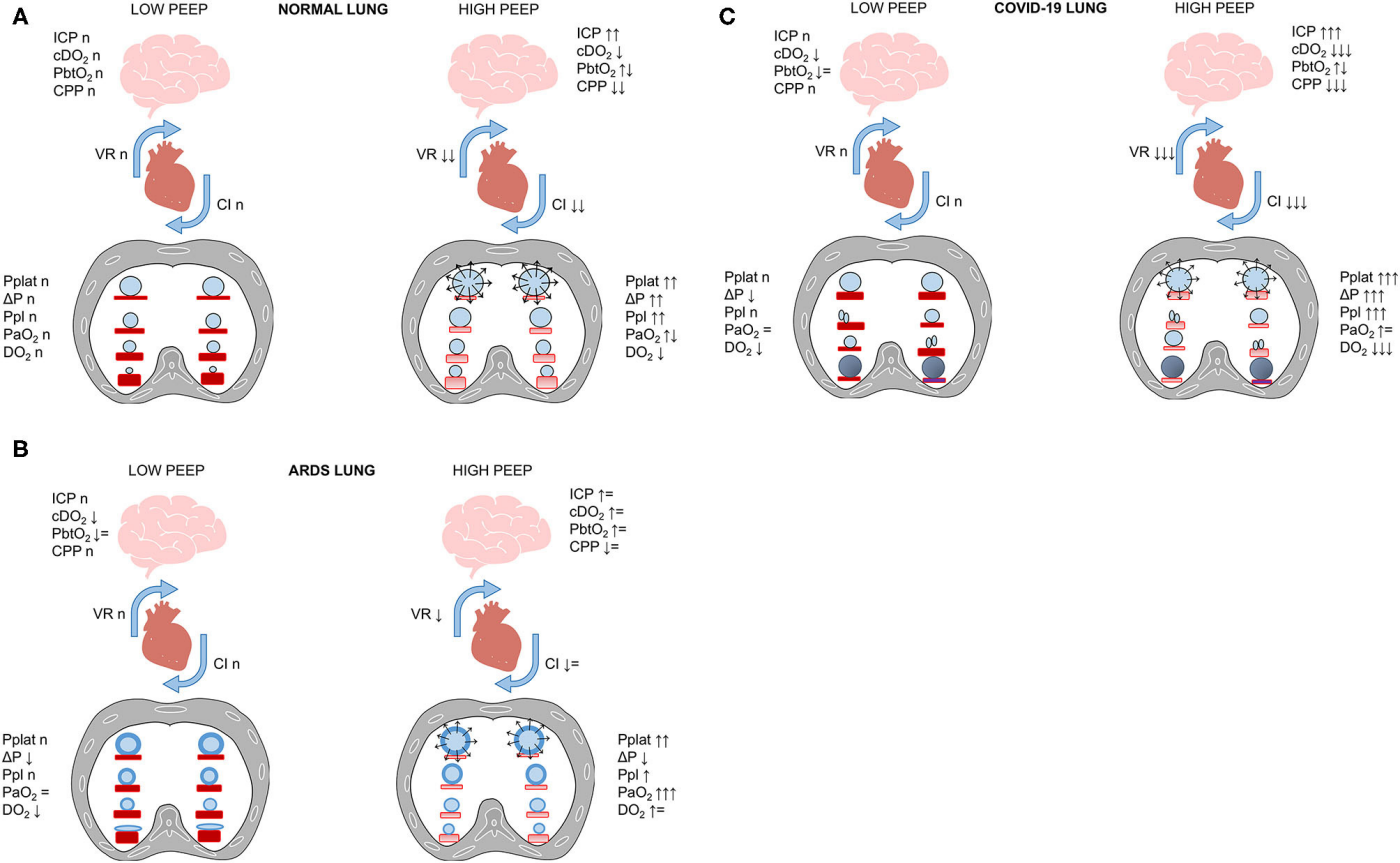
**(A–C)** Brain–lung–heart cross talk. SARS-CoV-2 lung infection can require mechanical ventilation, which heightens the pro-inflammatory cascade. In this figure, we propose the effect of increased PEEP on the cardiovascular system and CNS in healthy subjects **(A)**, ARDS **(B)**, and COVID-19 **(C)**. In normal lungs **(A)**, high PEEP and alveolar hyperdistention cause increased plateau pressure (Pplat), driving pressure (ΔP), and pleural pressure (Ppl), with consequent reduction of venous return (VR) and cardiac index (CI) and reduced cerebral perfusion pressure (CPP) and increased intracranial pressure (ICP). This can be partially offset by the presence of preserved gas exchange. In ARDS patients **(B)**, the increase in PEEP with recruitment of collapsed areas does not cause significant changes in hemodynamics or cerebral function, and can increase oxygen delivery (cDO_2_). Conversely, in COVID-19 patients **(C)** who do not respond to recruitment, the concomitance of alveolar hyperdistention after PEEP increase and hypoxemia can cause serious impairment of cerebral dynamics and cerebral hypoxemia (low PbtO_2_).

A correlation between acute lung injury and brain hypoxia has been described by Oddo et al. (89). Reduced systemic oxygenation may affect brain tissue oxygenation, thus leading to secondary brain damage. Measurement of brain tissue oxygenation tension (PbtO_2_) has confirmed that this parameter is strongly correlated with systemic oxygenation and markers of lung function, including partial pressure of carbon dioxide (PaCO_2_) and mean arterial pressure. Accordingly, impaired partial pressure of oxygen (PaO_2_)/fraction of inspired oxygen (FiO_2_) ratio has been associated with lower PbtO_2_ (89). In another study, patients who underwent an oxygen challenge with 100% FiO_2_ showed higher PbtO_2_ (90). Hypoxic–ischemic damage is also associated with impaired outcome (91). We believe this phenomenon should be considered one of the main mechanisms implicated in neurological dysfunction following SARS-CoV-2 infection. In fact, given these respiratory characteristics, “silent” hypoxia with normal/hypercapnic respiratory failure can occur due to compromised alveolar gas exchange (92). Our knowledge concerning hypobaric hypoxia can be derived from aviation medicine (93). High altitude correlates with severe hypoxemia, which triggers the carotid chemoreceptors, activating the respiratory drive; hypocapnia ensues. The oxyhemoglobin dissociation curve shifts to the left in response to respiratory alkalosis and increased affinity of oxygen for hemoglobin, thereby increasing the alveolar oxygen tension and systemic saturation after reducing alveolar carbon dioxide tension, as explained by the Bohr effect—the greater the acidity, the more carbon dioxide is eliminated (94). PaO_2_ and oxygen delivery (DO_2_) can be optimized by modulating blood pH and PaCO_2_, hemoglobin concentration, cardiac output, and arterial content of oxygen. These factors mean close attention is warranted when implementing lung-protective strategies, particularly when using low oxygen targets (55–80 mmHg) and permissive hypercapnia. In phenotype 1, characterized by lower potential alveolar recruitability, raising hemoglobin and cardiac output should be considered as a strategy to improve DO_2_, as explained in Figure 4. One possible side effect of higher hemoglobin is increased blood viscosity, raising the risk of cerebrovascular events (95). In phenotype 3 (ARDS-like COVID), prone positioning, higher PEEP, and RMs should be attempted instead to increase PaO_2_ and control PaCO_2_ levels. At this point, it is crucial that brain–lung–hemodynamics crosstalk be addressed (Figures 5A–C) (96). Current knowledge on the cerebral effects of mechanical ventilation has shifted in favor of moderate-PEEP strategies instead of low- or zero-PEEP strategies, due to possible beneficial effects on brain tissue oxygenation (97–99). Nevertheless, higher PEEP levels may be considered in COVID-19 phenotype 3 to reach acceptable levels of oxygen saturation in the brain (100), thus improving cerebral blood flow and perfusion (101). In this phenotype (but not in phenotypes 1 or 2), lung recruitment maneuvers might also improve oxygenation by improving gas exchange, although their effects on intracranial pressure (ICP) could be detrimental due to impaired jugular venous outflow and venous return (102).

According to the “blast injury theory,” the sympathetic storm, cytokine storm, and hyperinflammatory state caused by infection can induce a transient increase in intravascular pressure, with endothelial damage, raised pulmonary vascular hydrostatic pressure, and increased capillary permeability, thus promoting lung derangement and a secondary brain insult (103). This could explain, at least in part, why patients with severe COVID-19 have worse neurological outcomes (104). Both oxygen and carbon dioxide have been considered important determinants of cerebral homeostasis, due to their effects on cerebral blood flow (105). Low cerebral blood flow due to low PaCO_2_ is associated with cerebral ischemia, while high cerebral blood flow results in cerebral hyperemia and higher ICP (105). A rise in ICP may also be achieved by increasing PaCO_2_ if intracranial compliance is reduced. In patients not amenable to alveolar recruitment maneuvers, such as those with COVID-19 phenotype 1, overdistension of alveolar areas contributes to a rise in PaCO_2_ due to the increase in dead space, followed by cerebral vasodilatation. Conversely, in patients responsive to recruitment maneuvers (COVID-19 phenotype 3), shunt is reduced, oxygenation improves, and the PaCO_2_ is decreased, with lower dead space and less changes in ICP and cerebral perfusion (83, 106). PaO_2_, PaCO_2_, pH, hemoglobin, and DO_2_ might all be considered as clinical targets for bedside monitoring where available, to protect both the brain and the lung.

### Experimental and Clinical Evidence

The first report of brain autopsies in COVID-19 patients was published on June 12, 2020. Impressively, the authors reported that, at histologic analysis, all 18 examined patients (100%) had evidence of acute hypoxic ischemic damage to the cerebrum and cerebellum. Neither encephalitis nor any evidence of specific viral invasion was identified (107). The neuroimaging features of 108 hospitalized COVID-19 patients demonstrated a non-specific pattern, with predominance of acute ischemic infarcts and intracranial hemorrhage. MRI findings included the posterior reversible encephalopathy syndrome (PRES), hypoxic–ischemic encephalopathy, and exacerbation of preexisting demyelinating disease, corroborating the role of a hyperinflammatory/hypercoagulable state and brain–lung crosstalk as major mechanisms potentially underpinning neurological complications in COVID-19 (108). Further evidence of neurological involvement is the higher incidence of ICU delirium in COVID-19 patients when compared to non-COVID patients (26.8 vs. 7.7%, *p* = 0.003) (109). This may be explained by the fact that profound hypoxia is known to predispose to long-term cognitive impairment and hypoxic delirium phenotypes, whether caused by BBB dysfunction, inflammation, hypoperfusion, hypoxemia, or a combination thereof (110–112).

In summary, encephalopathy and cerebrovascular disease are the main neurological features identified in severe COVID-19 (73, 113). Despite compelling evidence of viral neurotropism, we believe this is not the primary causative factor of neurological involvement. Instead, in most cases it is likely due to impairment of the delicate equilibrium between the brain and the lung and to the hyperinflammatory, pro-coagulative state that is characteristic of SARS-CoV-2 infection.

## Conclusions

In COVID-19 patients, central and peripheral nervous system changes may be caused by viral neurotropism (such as impairment of olfaction and taste), by a hyperinflammatory and hypercoagulative state, or even by mechanical ventilation-associated impairment. Three distinct phenotypes of pulmonary injury have been identified in association with COVID-19 pneumonia, each requiring individualized respiratory support strategies to minimize lung injury and optimize oxygen delivery to different organs—including the brain. Data from prospective observational studies, randomized clinical trials, and autopsies are urgently needed to confirm the latest findings concerning the causal roles of hypoxic–ischemic brain damage, inflammation, and hypercoagulability in the neurological manifestations of COVID-19.

## Author Contributions

DB: design and conceptualization, drafting, and revising the manuscript for intellectual content. PA, PF, and PP: drafting and revising the manuscript for intellectual content. IB, GZ, LB, NP, DG, AV, MB, and AS: revising the manuscript for intellectual content. PP, PR, and CR: design and conceptualization and revising the manuscript for intellectual content. All authors: read and approved the final version of the manuscript.

## Conflict of Interest

Outside the submitted work DG reports honoraria from Stepstone Pharma GmbH and unconditional grants from MSD Italia and Correvio Italia. Outside the submitted work, MB has received funding for scientific advisory boards, travel, and speaker honoraria from Angelini, Astellas, AstraZeneca, Basilea, Bayer, BioMerieux, Cidara, Correvio, Cubist, Menarini, Molteni, MSD, Nabriva, Paratek, Pfizer, Roche, Shionogi, Tetraphase, Thermo Fisher, and The Medicine Company. The remaining authors declare that the research was conducted in the absence of any commercial or financial relationships that could be construed as a potential conflict of interest.

## Abbreviations

ACE2: angiotensin-converting enzyme-2
ANE: acute necrotizing encephalopathy
ARDS: acute respiratory distress syndrome
BALF: bronchoalveolar lavage fluid
BBB: blood brain-barrier
CA: Ammon's horn
CD: cluster of differentiation
CI: confidence interval
CNS: central nervous system
CoV: coronavirus
COVID-19: coronavirus disease 2019
CT: computed tomography
CXCR: chemokine receptor
DIC: disseminated intravascular coagulation
DO_2_: oxygen delivery
DPP4: dipeptidyl dipeptidase-4
ECMO: extracorporeal membrane oxygenation
FiO_2_ fraction of inspired oxygen
FOX: forkhead box
HLH: hemophagocytic lymphohistiocytosis
ICAM: intracellular adhesion molecule
ICH: intracerebral hemorrhage
ICP: intracranial pressure
IFN: interferon
MERS: Middle East respiratory syndrome
MHV: mouse hepatitis virus
MRI: magnetic resonance images
nCoV: novel coronavirus
OR: odds ratio
PaCO_2_: partial pressure of carbon dioxide
PaO_2_ partial pressure of oxygen
PbtO_2_ brain tissue oxygenation tension
PCR: polymerase chain reaction
PEEP: positive end-expiratory pressure
PRES posterior reversible encephalopathy syndrome
RM: recruitment maneuvers
RNA: ribonucleic acid
SARS: severe acute respiratory syndrome
TLRs: toll-like receptor
TMPRSS2 transmembrane serine protease 2
TNF: tumor necrosis factor
WHO: World Health Organization.
